# Study of curling mechanism by precision kinematic measurements of curling stone’s motion

**DOI:** 10.1038/s41598-022-19303-4

**Published:** 2022-09-03

**Authors:** Jiro Murata

**Affiliations:** Department of Physics, Rikkyo University, Tokyo, 171-8501 Japan

**Keywords:** Applied physics, Surfaces, interfaces and thin films

## Abstract

Why do curling stones curl? That is a question physicists are often asked, yet no answer has been established. Stones rotating clockwise curl right, contrary to our naive expectations. After a century of debate between contradicting hypotheses, this paper provides a possible answer based on experimental evidence. A digital image analysis technique was used to perform precision kinematic measurements of a curling stone’s motion to identify the curling mechanism. We observed a significant left–right asymmetric friction due to velocity dependence on the friction coefficient. Combined with the discrete point-like nature of the friction between ice and stone, swinging around slow-side friction points has been concluded as the dominant origin of the curling. Many new angular momentum transfer phenomena have been found, supporting this conclusion.

## Introduction

As one of the Winter Olympics events, the curling competition is attracting more and more attention. Along with the fun of the sport, there has been a lot of discussion about why the curling stone’s trajectory bends, i.e., curls, just like the question of the principle of a breaking ball in baseball or lift of airplanes. The curling’s mysterious behavior piques the public’s interest because of its opposite direction from the naively expected curling direction, considering the friction at the front edge. For almost a century, physicists have attempted but not succeeded in explaining the curling mechanism^1–7^. Not only that, but the situation is fraught with conflicting models, owing primarily to a lack of sufficient precise observation data.

**Figure 1:**
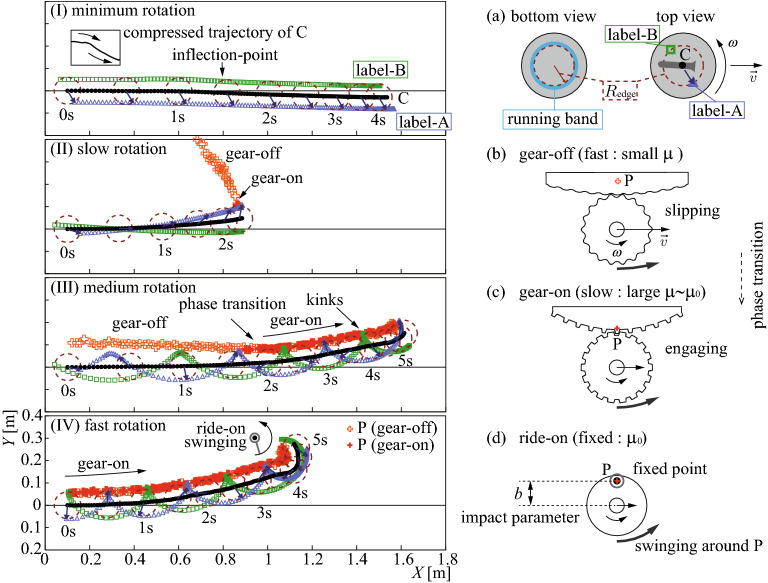
(**I**–**IV**) Examples of the curling stone’s trajectories for labels A, B, and C in the horizontal plane plotted for every $\Delta t$ (but $15\Delta t$ for the dotted circles and arrows). See (**a**) and Fig. 3 for the definitions. Raw A, B positions at $R=78\,{\mathrm{mm}}$ were corrected to be placed at $R_{\mathrm{edge}}=60\,{\mathrm{mm}}$. We obtained data sets with initial conditions of (**I**) $(|\omega _0|<0.3\,{\mathrm{rad/s}})$ for 18 low-speed $( v_0=0.3\,{-}\,0.7\,{\mathrm{m/s}})$ and 20 high-speed $(0.7\,{-}\,1.2 \,{\mathrm{m/s}})$ shots, (**II**) $(0.6\,{-}\,1.5\,{\mathrm{rad/s}})$ for 19 low-speed shots, (**III**) $(2\,{-}\,5\,{\mathrm{rad/s}})$ for 47 low-speed shots, and (**IV**) $(6\,{-}\,9\,{\mathrm{rad/s}})$ 18 low-speed shots. Gear-on (off) is defined as $R_{\mathrm{rot}} \equiv \overline{\mathrm{C}}{\mathrm{P}}\le (>) \,65\,{\mathrm{mm}}$. See Supplementary video online for motion capturing movie (tracking raw label A). (**a**–**d**) Stone’s geometry, interpretations of “gear-on/off, ride-on” phases.

Uniform friction over the bottom of curling stones cannot produce any systematic transverse momentum transfer. Therefore, possible hypotheses must include forward–backward asymmetry^5–8^ or left-right asymmetry^1,9^ of the friction strength. In addition, surface roughness is often highlighted to be necessary, which may cause discrete frictioning such as pivoting due to pebble structures on ice^10–14^ and dust and scratching on ice by the stone’s rough bottom surface^15^. If we suppose the Coulomb friction law (the dynamic friction force must be opposite to the velocity direction), the left-right asymmetry of the continuum friction cannot transfer longitudinal to the transverse momentum^7^. For this reason, many hypotheses recently proposed are based on the forward-backward asymmetry requesting stronger friction at the back edge^16–22^, or a creative idea of scratch-guide mechanism^23–27^, but none of which are established.

## Methods

A precision trajectory measurement, including the rotation degree of freedom, was performed to begin a data-driven model-independent discussion. A digital image analysis technique, originally developed as an optical alignment system for a high-energy accelerator experiment^28^ and as a displacement sensor for table-top gravity experiments^29,30^, was used.

The measurement was performed at Karuizawa Ice Park in Nagano. The stone’s positions were measured with a sub-millimeter resolution for each static video frame ($1920\times 1080$ pixels) obtained at $29.97\,{\mathrm{frames/s}}$ using a camera set on the top view position at $1800\,{\mathrm{mm}}$ above the ice surface. Positions of two labels A and B attached on the top surface of the stone (Fig. 1a) were measured for each frame with a time step of $\Delta t=1/29.97 \,{\mathrm{s}}$. Then, positions of A and B and their center C were obtained after radial position and parallax correction as vertically projected positions on the ice plane. The *XY* coordinates were defined relative to the direction of the initial velocity. $t=0$ was locally defined as the starting timing for each shot. The resolution of the image analysis system was $\sigma _x=47\; \upmu {\mathrm{m}}$ which was evaluated as the standard deviation of measured C’s position *x*, obtained in a dedicated measurement using a static stone. Similarly, resolutions of C’s velocity *v*, acceleration *a*, and angular velocity $\omega$ around C were obtained as $\sigma _v=2.0\; {\mathrm{mm/s}}$, $\sigma _a=47 \;{\mathrm{mm/s}}{^2}$ , and $\sigma _\omega =20 \;\mathrm {m rad/s}$, respectively. Here, *v*, $\omega$, (*a*) were estimated by comparing two (three) sequencial frames separated with $\Delta t$. The systematic (non-random) uncertainty, including non-linearities in the calibration, was $1.1\;{\mathrm{mm}}$, which might affect absolute value determinations. However, the corresponding relative systematic error was less than 1%, which can be regarded as negligible in the following discussion. The actual motion was disturbed by the messy vibration caused by the pebbles. All shots were made toward the $X>0$ direction with counter-clockwise rotation, but their initial conditions were not precisely controlled because we made them manually. Instead, they were measured. The stone’s mass and moment of inertia were $m=18.53\pm 0.04\,{\mathrm{kg}}$ and $I=0.15 \pm 0.02\,{\mathrm{kg}}{\mathrm{\,m}}{^2}$ around the center axis, respectively.

**Figure 2:**
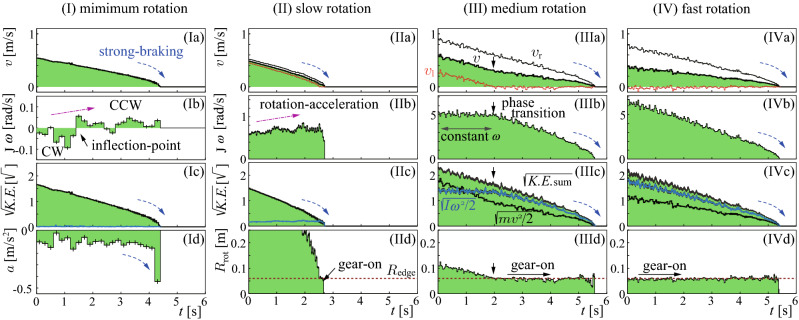
Time sequences for the same shots in Fig. 1, of the kinematic variables; C’s velocities *v*(*t*), C’s accelerations *a*(*t*), angular velocities $\omega (t)$, kinetic energies *K*.*E*., and $R_{\mathrm{{rot}}}(t)$. The square roots of *K*.*E*. were plotted for the translational components $mv^2/2$, the rotational components $I\omega ^2/2$, and their sums $K.E._{\mathrm{sum}}$. Vertical error bars were drawn if they were not negligible. Gear-on, phase-transition, strong-braking, rotation-acceleration, inflection-point in Fig. 1I, and constant $\omega$ are shown where they appeared.

## Results

**Figure 3:**
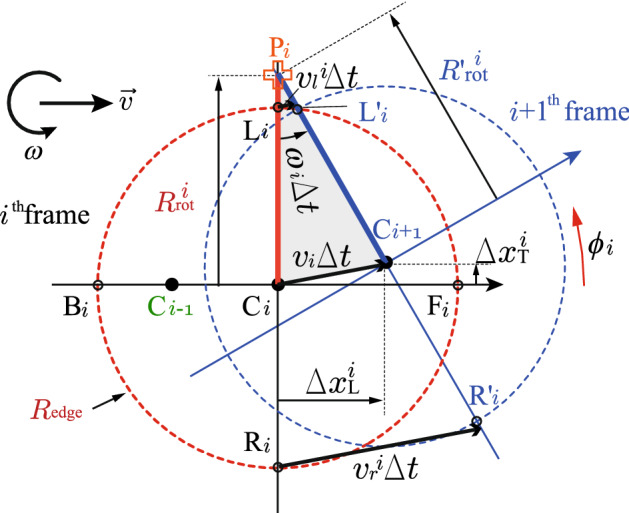
Parameter configurations. *i* denotes the $i{\mathrm{th}}$ frame at $t_i=i\Delta t$. The forward position ${\mathrm{F}}_i$ is determined on line $\overline{{\mathrm{C}}_{i-1} {\mathrm{C}}_i}$, then, $\phi _i$ is locally defined on the stone’s frame in CCW from this direction. ${\mathrm{L}}_i$, ${\mathrm{R}}_i$, ${\mathrm{F}}_i$, and ${\mathrm{B}}_i$ were set on $R_{\mathrm{{edge}}}$. ${\mathrm{P}}_i$ is defined as the intersection of lines $\overline{{\mathrm{C}}_i {\mathrm{L}}_i}$ and $\overline{{\mathrm{C}}_{{i+1}} {\mathrm{L'}}_i}$. Note that ${\mathrm{L'}}_i$ is the same position as ${\mathrm{L}}_i$ on the stone’s local frame, but that in $t=t_{i+1}$ on the ice’s frame. The “swinging” arm length is defined as $R_{\mathrm{{rot}}}^i = \overline{{\mathrm{C}}_i{\mathrm{P}}_i}=\Delta x_{\mathrm{{L}}}^i / {\mathrm{tan}} \Delta \theta _i+ \Delta x_{\mathrm{{T}}}^i$, and ${R'}_{\mathrm{rot}}^i= \overline{{\mathrm{C}}_{i+1}{\mathrm{P}}_i}=(\Delta x_{\mathrm{{L}}}^i / {\mathrm{tan}} \Delta \theta _i) / {\mathrm{cos}} \Delta \theta _i$, where $\Delta \theta _i=\omega _i \Delta t$. The velocities of ${\mathrm{L}}_i$ and ${\mathrm{R}}_i$ positions were approximately estimated as $v_{l(r)}^i=v_{i}\cdot [R_{\mathrm{{rot}}}^i-(+)R_{\mathrm{{edge}}}]/R_{\mathrm{{rot}}}^i$. $\omega _i$ were obtained from the label’s relative angular changing.

Figures 1, 2, and 3 show the trajectories, the time sequences of the kinematic variables for the same four typical shots, and parameter configurations. ${\mathrm{P}}$ is defined in Fig. 3, from which we can approximately determine the swinging center. Indeed, ${\mathrm{P}}$ exactly acts as the swinging center for a case of pure rotation around a fixed friction point on the left side position. We can determine ${\mathrm{P}}$ uniquely, which is defined as the intersection of two straight lines. In cases with no static friction points, ${\mathrm{P}}$ can be interpreted as the virtual swinging center of the stone, which area is extended outside the actual stone volume. Especially, $R_{\mathrm{rot}}\equiv \overline{\mathrm{CP}}\rightarrow \infty$ for pure translation with no rotation cases. Therefore, we can use $R_{\mathrm{rot}}$ as a quantity representing how static the left side friction is.

The most symbolic phenomenon among the obtained results was the strong swinging observed before stopping, as shown in Fig. 1IV “ride-on swinging.” The stone swung around an almost static left position ${\mathrm{L}}\cong {\mathrm{P}}$ on the radius of $R_{\mathrm{{edge}}}$ as a simple orbital rotation (Fig. 1d). Here $R_{\mathrm{edge}}$ was set to the inner radius of the “running band” (contacting ring) of the stone’s bottom (Fig. 1a).

Similar relatively strong curling was observed, as shown in Fig. 1II–IV “gear-on” phase. In this phase, positions of ${\mathrm{P}}$ were not static but drifted while maintaining $R_{\mathrm{rot}}\cong R_{\mathrm{{edge}}}$ positions (Fig. 1c). In Fig. 1III and IV, the velocities of ${\mathrm{L}}$ (i.e., $v_l$) were almost zero, as appeared as “kinks” of the label A, B’s trajectories. In fact, Fig. 2IIIa and IVa show $v_l\cong 0$ during $R_{\mathrm{rot}}\cong R_{\mathrm{edge}}$ gear-on phase (Fig. 2IIId and IVd). As shown in Fig. 1II and III, ${\mathrm{P}}$ moved from far distances to the $R_{\mathrm{edge}}$ position during the “gear-off” phase (Fig.1b) and then remained there stably after this “phase transition” to gear-on phase.

The minimum rotation shot also revealed interesting features. As shown in Fig. 2Ia, the deceleration rate of *v*(*t*) seems to be increasing at around the end ($t>4\;{\mathrm{s}}$), suggesting the existence of velocity dependence of the friction coefficient $\mu$. In fact, *a*(*t*) showed a large value just before the end, implying the strong-braking, as shown in Fig. 2Ia and Id. At the end of this section, we will discuss the significance of this behavior of $\mu (v)$ in a statistical analysis. In Fig.2Ib and Id, *a*(*t*) and $\omega (t)$ were shown by rebinning the time sequence combining $8\Delta t$ to suppress fluctuations, which resolutions were $\sigma _a^{8\Delta t}=17\;{\mathrm{mm/s}}{^2}$ and $\sigma _\omega ^{8\Delta t}=7.1 \;{\mathrm{mrad/s}}{^2}$. $|\omega (t)|$ was small, but it can be highlighted that the rotation direction was significantly transitioned from CW (clockwise) to CCW (counter-clockwise) at $t\cong 1.4\,{\mathrm{s}}$ occasionally. This transition timing coincided with the timing of the “inflection-point”, which can be noticed if we carefully observe the compressed image of the trajectory shown in the inlet figure of Fig. 1I. It shows a transition of rightward to leftward curling. This result implies that $\omega$ was not simply decelerating but sometimes accelerating and that the changing of $\omega$ correlated with the curling. This “rotation-acceleration” phenomenon can also be found in Fig. 2IIb for the slow rotation shot.

The transitioning phenomena were also found for the translational motion. In Fig. 2IIIa “phase transition,” the deceleration rate of *v* suddenly decreased after $t\cong 2\,{\mathrm{s}}$. It was at the point that the gear-on phase began. At the same time, $\omega$ started a rapid deceleration (Fig. 2IIIb). This correlation can be well understood by checking the kinetic energies, *K*.*E*., as shown in Fig. 2IIIc. Their square roots were plotted to see quantities proportional to velocities. The translational and rotational components exhibited the transition, but their sum did not. This disappearance of the transition is particularly intriguing. It means that the total *K*.*E*. of a stone was conserved, except for the frictional loss while transferring the energy between translational ($\frac{1}{2} mv^2$) and rotational ($\frac{1}{2} I\omega ^2$) motions as

1 $$\begin{aligned} \frac{d}{dt}\left( \frac{1}{2}mv^2\right) +\frac{d}{dt}\left( \frac{1}{2}I\omega ^2\right) +{\mathrm{frictional\;loss\;rate}}=0, \end{aligned}.$$

But the total frictional loss rate in the system did not have the sudden change at the phase-transition timing. It is interesting to see the common features of strong-braking in the curves showing this velocity-dependent frictional deceleration at the timing just before the end of *v* in Fig. 2Ia, IIa, IIIa and IVa, $\omega$ in Fig. 2IIIb and IVb, and $\sqrt{E_{\mathrm{sum}}}$ in Fig. 2Ic, IIc, IIIc and IVc.

The gear-off phase was also interesting, representing a situation in the actual curling games. $\omega$ was almost constant during the gear-off phase, as shown in Fig. 2IIIb. The transfer of translational and rotational energies helps to explain this situation. The rotational energy was fed by the translational energy, preventing deceleration due to rotational friction loss (Fig. 2IIIc). Therefore, we should not simply interpret the observed stability of $\omega$ during the gear-off phase as a result of minimal rotational friction coefficient. In contrast, the rotational energy fed the translational energy, as shown in the deceleration relaxation of *v* shown in Fig. 2IIIa during the gear-on phase.

**Figure 4:**
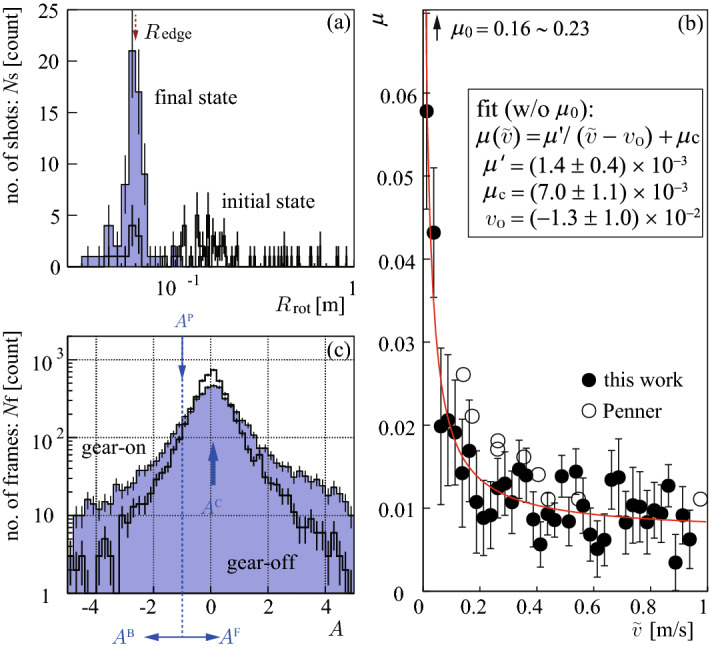
(**a**) $R_{\mathrm{rot}}$ distribution at the initial and final states with Poisson error bars, obtained for all 73 completely stopped shots, except for minimum rotation shots, (**b**) $\mu ({\tilde{v}})$ obtained using 38 minimum rotation shots, compared with Penner’s result^10^, (**c**) distribution of *A* for all frames of the shots in (**a**) with Poisson error bars.

All shots we measured were analyzed, not only for the typical shots shown in Figs. 1 and 2. $R_{\mathrm{rot}}$ distributions at initial and final states are plotted in Fig. 4a, with Poisson errors $= \sqrt{{sample\;number}}$. It can be confirmed that the convergence $R_{\mathrm{rot}}\rightarrow R_{\mathrm{edge}}$ was the common feature of all shots, independent of the initial conditions $(v_0,\omega _0)$, except for the minimum rotation cases. The observed peak of $R_{\mathrm{rot}}^{\mathrm{peak}}=58\,\pm \,1.3\,{\mathrm{mm}}$ for the final states was compared with the running band regions of $R_{\mathrm{band}}=65\pm 5\,{\mathrm{mm}}$. Then, their difference should be interpreted as the azimuthal angular distribution of the actual swinging center positions, which might be spread out at approximately $\pm {\mathrm{cos}}^{-1}(R_{\mathrm{rot}}^{\mathrm{peak}}/R_{\mathrm{band}})\cong \pm 27^\circ$ around $\phi =90^\circ$.

As shown in Fig. 2Id, $\mu$ seems strongly dependent on *v*. To confirm this, $\mu (v)$ might be estimated using the correlation between *v*(*t*) and $a(t)=\mu g$ for the minimum rotation shots. However, $\omega$ of the minimum rotation shots was not precisely zero. Therefore, we should have considered the deceleration of *v* caused by energy leakage from translation to rotation. ${\tilde{v}}=\sqrt{2 K.E._{\mathrm{sum}}/m}$ and ${\tilde{a}}=d{\tilde{v}}/dt$ were used after rebinning the time sequence combining $8\Delta t$ to suppress statistical fluctuations. Then, $\mu ({\tilde{v}})={\tilde{a}}/g$ was obtained as shown in Fig. 4b, using the local value of the gravitational acceleration of $g=9.796\,{\mathrm{m/s^{2}}}$. Here, mean values and their standard errors $({standard\; deviation}/\sqrt{sample\; number})$ of many ${\tilde{a}}$ samples from 38 minimum rotation shots, in the same ${\tilde{v}}$ bins, were used to obtain $\mu ({\tilde{v}})$ and their errors. The ${\tilde{v}}$ bin width of $25\;{\mathrm{mm/s}}$ was sufficiently larger than $\sigma _v$, and only data with ${\tilde{v}}>10 \;{\mathrm{mm/s}}$ were used.

The remarkable velocity dependence of $\mu$ was confirmed, showing a rapid increase before stopping. It meant that the strong-braking found in Fig. 2Ia and Id was confirmed as a common feature over all the samples in this statistical analysis. This is crucial to understand the curling mechanism, directly implying a friction enhancement on the slower side. This characteristic itself has been well known at least for a century^1^, as a result of ice melting. However, a reliable data set covering a very slow region at $v<0.1\;{\mathrm{m/s}}$ was newly obtained in this study. It owed to the high precision which enabled us to extend the sensitivity well down to $v\sim \sigma _v$. Figure 4b also shows the fitting result, which may be useful for future model calculations attempting to predict the curling trajectories as well as microscopic studies on the physics of friction. The static friction coefficient $\mu _{0}$ measured using a spring scale was also shown, which was not used for the fitting. By utilizing this $\mu (v)$ data with *m* and *I*, one may be able to build a simulation tool to predict a curling stone’s motion just by inputting $v_{0}$ and ${\omega _{0}}$.

## Discussion

Let us now attempt to understand the obtained results shown above. First, the strong curling, as shown in Fig. 1IV “ride-on swinging,” was a clear indication of the existence of strong point-like frictions. It should be caused by pivoting due to relatively large pebbles on ice^12^ or dust or scratching by the rock’s rough bottom^15^ or their accidental coincidences. Therefore, these phenomena occurred by chance, with less than 50% of our rotating shots exhibiting it.

The energy/momentum transfer between translational and rotational motions was found. This was observed as the accelerating rotation in Fig. 2Ib and IIb, as the deceleration relaxation of *v* in the gear-on phase in Fig. 2IIIa, and as the constant $\omega$ in Fig. 2IIIb. It also meant the transfer between the orbital-angular momentum (for revolution around a fixed position) *L* and the spin-angular momentum (for self-rotation) *S*^31^, conserving the total angular momentum $J=L+S$, if there is no external torque. For $L=mvb$ around a friction point and $S=I\omega$ (*b* is the impact parameter, i.e., the perpendicular distance between the path of an incident particle and the center of force. See Fig. 1d, the $L\leftrightarrow S$ transfer requires offset impact, i.e., $b\ne 0$. Therefore, the observed angular momentum transfer must be caused by point-like impacts at a non-zero net offset position $\langle b \rangle$. Any forward-backward asymmetric friction cannot produce such angular momentum transfer because $\langle b \rangle =0$. Angular momentum transfer due to an offset collision to a fixed point cannot avoid swinging. The swinging leads to the leftward curling if the impact point is at $90^\circ \lesssim \phi < 180^\circ$. This can be confirmed as a coincidence of the curling inflection and the $\omega$ change in the minimal rotation shot as shown in Figs. 1I and 2Ib.

The converging $R_{\mathrm{rot}}\rightarrow R_{\mathrm{edge}}$ (Fig. 4a) can be understood as the frictional force at ${\mathrm{L}}$ being always opposite to the $v_l$ direction, suppressing $|v_l|$. The observed converging $v_l\rightarrow 0$ (Fig. 2IIIa) also indicates that the friction was strongest at ${\mathrm{L}}$ around the $\phi$ direction in the running band. This backward friction at ${\mathrm{L}}$ assisted rotation when $v_l>0$ via the $L\rightarrow S$ transfer, preventing deceleration of $\omega$ during the gear-off phase. The stability of $v_l \cong 0$ and $R_{\mathrm{rot}} \cong R_{\mathrm{edge}}$ during the gear-on phase (Fig. 2IIIa,IVa,IIId,IVd) must be due to the large local static friction $\mu _0$ at ${\mathrm{L}}$. It prevented $|v_l|$ from enlargement by sequentially switching the engaging points by next-to-next, similar to engaging gears (Fig. 1c). The frictioning points for the gear-off phase were not static but dragged while scratching the ice, similar to slipping tires (Fig. 1b), which must be caused by the relatively large $\mu$ at small $v_l$.

The observed $\mu (v)$ (Fig. 4b) indicated that the probability of having discrete impacts was greater at ${\mathrm{L}}$ than at ${\mathrm{R}}$ because continuum friction is not a fundamental concept but only a result of an artificial coarse-graining (averaging) treatment for many real microscopic impacts. As a result, it should be concluded that the combination of 1. swinging around a discrete frictioning point on the ice (pivoting/scratching)^10–15^ and 2. the probability of the discrete frictioning is greater at the slow-side than at the fast-side because the velocity dependence of $\mu$^1,10^ should be the dominant curling mechanism. The convergence $v_l\rightarrow 0$ meant the existence of force to generate strong local static frictioning $\mu \rightarrow \mu _0$ at the slow-side, which worked as the “adhesive friction” requested in the pivoting models^10–12^.

*S* provided the left-right asymmetry of the swinging probability but was not typically the primary momentum source of the swinging. That was *L*, which was transferred from a straight motion with the impact parameter *b* to an orbital rotational motion with the arm length *b*, resulting in swinging for $v_l>0$, i.e., the slow rotating gear-off cases. *S* can directly contribute to the swinging via $S\rightarrow L$ transfer, but it was effective only for the fast rotation cases satisfying $v_l\le 0$. It should provide an answer to the known question^10,23^ of why the total amount of curl is not proportional to, and only weakly depends on, $\omega _0$, part of which can be seen by comparing Fig. 1II and III. On the other hand, $\omega _0$ dependence was becoming visible in the faster rotation cases due to the contribution from *S*^20^. In addition, deceleration relaxation of *v* due to $S\rightarrow L$ transfer during the gear-on phase (Fig. 2IIIa,IVa) should be the direct answer to why extremely fast rotating stones tend to travel further^10^. The stored intrinsic rotational energy should have been used to help the translational motion overcome friction.

In a real curling game, the brush-sweeping is effective not only for extending the stopping range by reducing $\mu$ but also for controlling the curling. Indeed, sweeping on the forward-right region leads to leftward curling. It is because of the reduction of the discrete frictioning on the right side. The unpredictable motion of the minimum rotation case is analogous to “knuckleball” in baseball, implying that a slight rotation should be preferred for a stable control to avoid random angular momentum transfer. The players should also remember that the occasional ride-on swinging phenomenon randomly affects the final stopping position.

Finally, the forward-backward asymmetry was examined. Although the inhomogeneous distribution of $\mu$ cannot be measured directly, we can estimate it because the discrete frictioning probability is proportional to $\mu$. A useful tool was comparing the lengths ${R}_{\mathrm{rot}}$ and ${R'}_{\mathrm{rot}}$ (See Fig. 3). By defining

2 $$\begin{aligned} A\equiv \frac{R'_{\mathrm{rot}}-R_{\mathrm{rot}}}{R'_{\mathrm{rot}}+R_{\mathrm{rot}}} \frac{4}{\Delta \theta ^2}-1, \end{aligned}.$$

**Figure 5:**
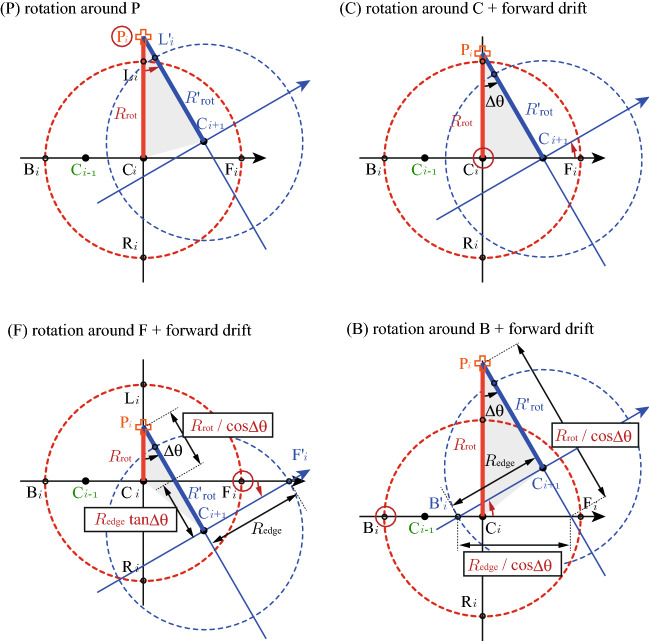
Relationship between $R_{\mathrm{rot}}$ and ${R'}_{\mathrm{rot}}$ for the typical cases. (**P**) Rotation around P: $R'_{\mathrm{rot}}=R_{\mathrm{rot}}$, i.e., $A^{\mathrm{P}}=-1$, (**C**) Rotation around C + forward drifting: $R'_{\mathrm{rot}}=R_{\mathrm{rot}}/{\mathrm{cos}}\Delta \theta$, i.e., $A^{\mathrm{C}}={\mathcal {O}}(\Delta \theta ^2)$, (**F**) Rotation around F + forward driting: $R'_{\mathrm{rot}}=R_{\mathrm{rot}}/{\mathrm{cos}}\Delta \theta +R_{\mathrm{edge}} {\mathrm{tan}}\Delta \theta$ , i.e., $A^{\mathrm{F}}\cong 2R_{\mathrm{edge}}/[R_{\mathrm{rot}}\Delta \theta ]-1 > -1$, (**B**) Rotation around B + forward driting: $R'_{\mathrm{rot}}=R_{\mathrm{rot}}/{\mathrm{cos}}\Delta \theta -R_{\mathrm{edge}} \mathrm{tan}\Delta \theta$ , i.e., $A^{\mathrm{B}}\cong - 2R_{\mathrm{edge}}/[R_{\mathrm{rot}}\Delta \theta ]-1 < -1$.

The swinging center positions were estimated. For example, $A^{\mathrm{P}}=-1$ for pure rotation around P without drifting, and $A^{\mathrm{C}}={\mathcal {O}}(\Delta \theta ^2)\sim 0$ for rotation around C with forward drifting. For rotation around F (B) with forward driting, $A^{\mathrm{F (B)}}>(<)-1$, which means that a large value of *A* implies swinging around F rather than B. Details of the estimation of ${R'_{\mathrm{rot}}}$ to obtain *A* for (P, C, F, B) are shown in Fig. 5.

Mean values and their standard errors of *A* for all frames in 73 completely stopped shots, except for minimum rotation shots, were obtained as $\langle A \rangle =0.149\pm 0.020$ (gear-on), $=0.056\pm 0.013$ (gear-off), showing positive $\langle A \rangle$. Figure 4c shows the *A* distributions with Poisson errors, which were dominated by $A^{\mathrm{C}}$. The realistic left side swinging with forward drifting should be distributed in $A^{\mathrm{P}}<A< A^{\mathrm{C}}$, but it was not visible. This must be because the probability of causing $A=A^{\mathrm{P}}$ is relatively negligible to $A=A^{\mathrm{C}}$. A slight asymmetry enhancing right side with respect to $A=0$ can be noticed, indicating that $A=A^{\mathrm{F}}$ was preferred to $A=A^{\mathrm{B}}$. Indeed, asymmetries of the integration of Fig. 4c between $A>0$ and $A<0$ were obtained with Poisson errors as $(2.03\pm 0.14)\times 10^{-1}$ (gear-on), $= (3.13\pm 0.14)\times 10^{-1}$ (gear-off). This asymmetry corresponds the positive $\langle A \rangle$. The dominant $A^{\mathrm{C}}$ contribution should have had symmetric shapes to $A=0$, and $A^{\mathrm{P}}$ must have enhanced $A<0$. Thus, the observed positive $\langle A \rangle$ meant that the $A^{\mathrm{F}}\text {-}A^{\mathrm{B}}$ asymmetry was significantly positive.

This result indicated that $\mu ({\mathrm{front}})>\mu ({\mathrm{back}})$, which is naively acceptable but may lead to cause opposite curling. Therefore, this effect should suppress the major curling, which can provide another possible reason for the weak dependence of the total curl on $\omega _0$. However, considering that the sensitivity of using *A* on probing frictioning points is not quantitatively known, we should interpret the observed statistically significant positive $A^{\mathrm{F}}\text {-}A^{\mathrm{B}}$ asymmetry as that, we found no evidence for the unnaturally large negative asymmetry indicating $\mu ({\mathrm{front}})\ll \mu ({\mathrm{back}})$ requested by the previously proposed forward-backward asymmetry models^5–8,16–22^ in this method.

## Conclusion

In conclusion, it has been found that swinging around the discrete left-right asymmetric frictioning points is the dominant curling mechanism. Each part of this conclusion is not perfectly new, repeatedly suggested as attractive hypotheses in previous works^1,10–15^. Except for $\mu (v)$, most of the featured rich results supporting the conclusion presented here are new, indicating the angular momentum transfer and the point-like nature of friction. Future model calculations must reproduce not only curling trajectories but also the phase transition and other angular momentum transfer phenomena. This work does not propose a hypothesis but presents the principle of curling based on the model-independent experimental evidence to solve this “mystery of the century.”

## Supplementary Information

Supplementary Video.

## Data Availability

The datasets generated during and analyzed during the current study are available from the corresponding author on reasonable request. Especially, the trajectory data will be useful to compare with simulation studies.
